# The Effect of Whole-Diet Interventions on Memory and Cognitive Function in Healthy Older Adults – A Systematic Review

**DOI:** 10.1016/j.advnut.2024.100291

**Published:** 2024-08-19

**Authors:** Lina Tingö, Cecilia Bergh, Julia Rode, Maria Fernanda Roca Rubio, Jonas Persson, Linnea Brengesjö Johnson, Lotte H Smit, Ashley N Hutchinson

**Affiliations:** 1 Nutrition-Gut-Brain Interactions Research Center, School of Medical Sciences, Örebro University, Örebro, Sweden; 2 Department of Biomedical and Clinical Sciences, Division of Inflammation and Infection, Linköping University, Linköping, Sweden; 3 Food and Health Program, Örebro University, Örebro, Sweden; 4 Clinical Epidemiology and Biostatistics, Faculty of Medicine and Health, Örebro University, Örebro, Sweden; 5 School of Health Sciences, Faculty of Medicine and Health, Örebro University, Örebro, Sweden; 6 School of Behavioral, Legal, and Social Sciences, Örebro University, Örebro, Sweden; 7 Aging Research Center (ARC), Karolinska Institute and Stockholm University, Stockholm, Sweden

**Keywords:** elderly, prevention, cognitive decline, nutrition, memory, diet

## Abstract

An increasing number of cross-sectional studies suggests that diet may impact memory and cognition in healthy older adults. However, randomized controlled trials investigating the effects of whole-diet interventions on memory and cognition in healthy older adults are rather rare, and conflicting results are often reported. Therefore, a systematic review was conducted to compile the current evidence regarding the potential effects of whole-diet interventions on *1*) memory and *2*) other cognitive outcomes in older adults. Studies that reported on randomized controlled trials with dietary interventions in healthy older adults (≥60 y) were included. Studies utilizing supplements, single food items, or trials in specific patient groups (i.e., neurodegenerative diagnoses) were excluded. For the 23 included articles, the main outcomes examined fell into 1 or more of the following categories: cognitive task-based outcomes related to memory, other cognitive task-based outcomes, and additional outcomes related to cognitive function or disease risk. Three of the studies that investigated dietary interventions alone and 2 multidomain studies showed positive effects on memory function, whereas 5 multidomain interventions and 1 intervention that focused on diet alone showed positive effects on other cognitive outcomes. The effect of randomized, controlled whole-diet interventions on memory and cognitive function in healthy older adults is modest and inconclusive, highlighting the need for more well-designed, sufficiently powered studies. Furthermore, the potential mechanisms by which diet impacts cognition in healthy aging need to be elucidated.

This systematic review is registered in PROSPERO as CRD42022329759.

**Statements of Significance**

This systematic review is the first to specifically focus on the effects of whole-diet interventions on memory in healthy older adults. While revealing modest effects on memory, analysis of the included randomized clinical trials highlights the need for more well-designed, sufficiently powered studies in this field.

## Introduction

As life expectancy in developed countries continues to increase, the population of adults ≥60 y is expected to grow by 56% in the next 15 y [1]. This “global aging” phenomenon will be associated with increases in aging-related disease and disability, particularly impacting the prevalence of memory loss and cognitive decline [2]. Ranging from mild cognitive impairment (MCI) to Alzheimer’s disease and other dementias, decline of memory function exerts significant economic, societal, and personal burden. This impending global increase in memory loss and overall cognitive decline has actualized an interest in identifying lifestyle-related factors to prevent and reduce these disorders.

Increasing evidence suggests that a lifestyle-related factor that potentially has a significant impact on memory and cognitive function is diet [[3], [4], [5], [6]]. Findings from several studies suggest that certain food products and supplements such as ω-3 polyunsaturated fatty acids [7], walnuts [8], berries, and flavonoids [9] are associated with improved memory and cognitive performance. For example, Ortega et al. [10] investigated if there are associations between food consumption and cognitive performance in a cohort of older adults without cognitive impairment. They found that higher scores on the Mini-Mental State Examination (MMSE), an instrument assessing certain aspects of memory and cognitive function, were associated with lower intake of unsaturated fatty acids, saturated fatty acids, and cholesterol as well as higher intake of total food, fruit, carbohydrates, thiamine, folate, and vitamin C than lower scores. These results suggest that the consumption of specific nutrients or an overall healthier diet may contribute to an improved memory and better cognitive function in older adults. In addition, a prospective cohort study [11] examined the association between adherence to a Mediterranean dietary pattern [i.e., the Mediterranean-Dietary Approaches to Stop Hypertension (DASH) Intervention for Neurodegenerative Delay (MIND) diet] and cognition. The study found that adherence to the MIND diet was associated with a better verbal memory score. In addition, McEvoy et al. [12] evaluated the association between 2 Mediterranean diets and cognition in a nationally representative population in the United States. In this population-based, cross-sectional study, they observed that greater adherence to either the Mediterranean diet (MedDiet) or the MIND diet was associated with better cognitive performance, specifically in the domains of episodic memory, working memory and attention. Furthermore, adherence to the MIND diet has been shown to reduce Alzheimer’s disease and dementia risk [13,14], as well as decrease risk and slow the progression of parkinsonism in older adults [15].

Together, these findings suggest that certain dietary patterns are associated with improved memory function in older adults. However, to investigate whether changes in diet affect memory and cognitive function in this population, well-designed, randomized, and controlled trials need to be performed. To date, there are a few systematic reviews summarizing the findings of such trials using specific supplements and food products [16,17] and 2 more recent publications summarizing the effects of whole-diet interventions (i.e., dietary interventions including a whole meal plan or intention to intervene on the habitual way of eating) on general cognition or cognitive decline [18,19]. We recognized a need to complement this field with a systematic, comprehensive, and unbiased summary of prospective randomized trials with whole-diet interventions (alone or in combination with other interventions) and their potential to impact the memory of older adults. Here, we have compiled information on study design, specific outcomes, study quality, and risk of bias to explore the current evidence on whether whole-diet interventions are a viable nonpharmacological strategy to improve or maintain cognitive function in older adults, with a specific focus on *memory*.

## Methods

### Protocols and registration

This systematic review is registered in PROSPERO as CRD42022329759. Details of the systematic review were initially submitted to PROSPERO on 3 June 2022. The registration record was automatically published after undergoing basic automated checks for eligibility and was formally registered on 14 June 2022.

### Information sources and search strategy

A comprehensive search specifically tailored to find randomized trials investigating memory outcomes was conducted by a medical research librarian in the following electronic bibliographic databases: Medline, Embase.com, Web of Science Core Collection (Editions = A&HCI, ESCI, CPCI-SSH, CPCI-S, SCI-EXPANDED, SSCI), and Cochrane Library. To limit the review to the most current studies, the search was restricted to articles in English published in peer-reviewed scientific journals in English published in peer-reviewed scientific journals during the last 20 y. Conference abstracts in Embase were excluded, in accordance with the inclusion criteria. The complete search strategy applied in the different databases is available in Supplementary Material S1. All records identified in the search were imported into an EndNote library and duplicates were removed.

### Eligibility criteria

All randomized controlled trials (RCTs) that reported dietary interventions in healthy older adults (defined as age ≥60 y) and not conducted in a specific disease or diagnosis cohort were eligible for inclusion. Studies reporting interventions with supplements or single food items were excluded. The dietary intervention could, however, be given as part of a multidimensional protocol, such as combinations of dietary advice and physical activity or other lifestyle interventions, and still be included. Studies reporting clinical trials involving patient groups with a diagnosis of Alzheimer’s disease, dementia, or other neurological disease were excluded. However, studies including participants with symptoms suggestive of MCI were included. Gray literature, such as abstracts, theses, or commentary articles, were excluded.

### Study selection

Two pairs of researchers (AH, JR and LT, CB) independently screened the titles of the studies identified by the search strategy; the 2 pairs screened half of the titles in parallel, after which they switched records and double-checked the eligibility of the items suggested for inclusion by the other couple. The corresponding abstracts were then retrieved and divided between 4 of the co-authors (MFRR, JR, LS, LBJ) for eligibility screening, after which the entire research team came together to discuss the inclusion of abstracts for further screening of the full-text articles. Full texts of potentially eligible studies were subsequently divided between all co-authors and independently assessed for eligibility. The entire research team then came together again to decide on the final set of papers for extraction. The papers that were excluded after the full-text review and the reasons for exclusion are summarized in Supplemental Material S2.

### Data extraction

The Cochrane Data Collection Form for Intervention Reviews: RCTs only (version 3) was used for data extraction and quality assessment of the full-text articles, after which the authors came together to discuss the information retrieved and agreed on which papers to include. To ensure accuracy and completeness of the extracted information, the information from the included papers entered on the data collection form was then cross-checked by another member of the team. Finally, all data was gathered in a master file to create an overview of the included studies.

### Risk of bias and study quality assessment

All included studies were individually assessed regarding risk of bias in their design and reporting by the first author (LT). For this purpose, the Data Collection Form for Intervention Reviews: RCTs only (version 3, April 2014) was used, taking guidance from Higgins et al. [20] on the scoring strategy. The studies were evaluated based on their general execution, and all outcomes deemed as relevant for this review were weighted together.

The articles were scored on the following items:

• Random sequence generation
• Allocation concealment
• Blinding of participants and personnel
• Blinding of outcome assessment
• Incomplete outcome data
• Selective outcome reporting

The above items were scored as introducing Low, High, or Unclear risk of bias. Unclear risk of bias was used if there was insufficient detail about what happened in the study or if what happened in the study was known but the arising risk of bias was unknown. Low risk of bias was scored if there were no reasons to suspect any introduction of bias, whereas high risk of bias was used if there were firm reasons to suspect that bias may have been introduced. Before finalization, the risk of bias assessment was further reviewed by authors AH and CB.

## Results

**FIGURE 1:**
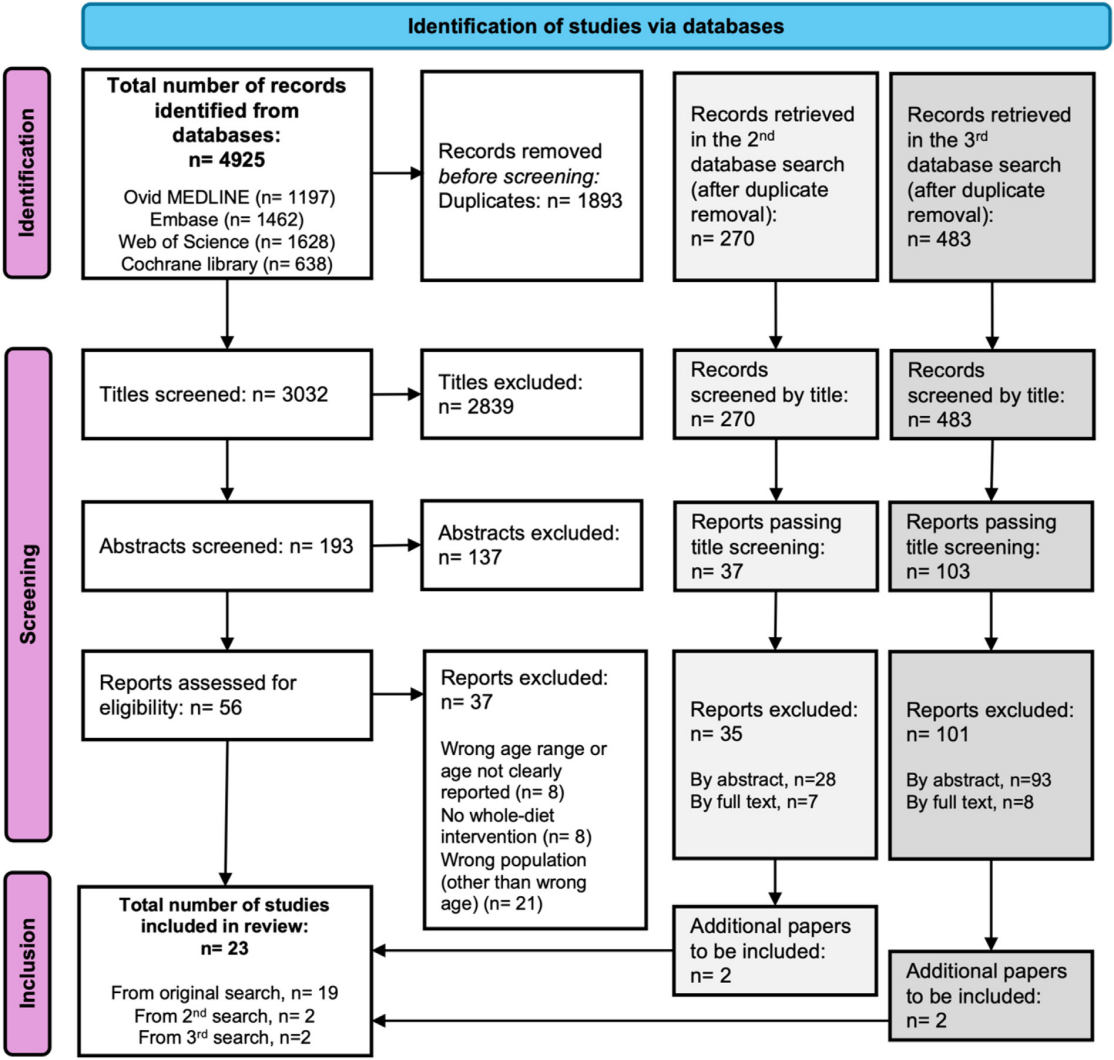
PRISMA flowchart (version 2020). PRISMA, Preferred Reporting Items for Systematic Reviews and Meta-Analyses.

This review includes 23 articles based on 13 RCTs reporting on the *1*) design or *2*) effect of whole-diet interventions on memory and other cognitive outcomes in healthy older adults [[21], [22], [23], [24], [25], [26], [27], [28], [29], [30], [31], [32], [33], [34], [35], [36], [37], [38], [39], [40], [41], [42], [43]]. The initial database search, commenced in spring of 2022, returned 3032 unique entries of which 19 were included in this review [[21], [22], [23], [24], [25], [26], [27], [28], [29], [30], [31], [32], [33], [34], [35], [36], [37], [38], [39]]. An updated search of the literature was conducted in January 2023 and returned 2 additional articles to be included in the final manuscript [40,41]. Finally, a third search conducted in May 2024 returned 2 articles to be included [42,43]. Figure 1 shows an overview of the selection process and Supplementary Material S1 details the full search strategy.

### Interventional design

Of the 13 studies, diet was part of a multidomain intervention in 8, whereas only 5 studies focused on the effects of a whole-diet intervention alone. Most included studies used some type of behavioral intervention: either multidomain [Multidomain Alzheimer Preventive Trial (MAPT) [[21], [22], [23]]; Finnish Geriatric Intervention study to Prevent Cognitive Impairment and Disability (FINGER) [[24], [25], [26], [27], [28], [29], [30], [31]]; Body, Brain, Life for Cognitive Decline (BBL-CD) [37]; eMIND [39], Johari et al. [34] and LIILAC [38]; Chatterjee et al. [40]; Osuka et al. [41]; and Sheffler et al. [42]] or dietary advice (MedLey [35], NU-AGE [36], Krikorian et al. [33], and MIND [43]). The exception was the Bayer-Carter et al. [32] study, which used an intervention in the form of meals delivered to the participant’s home without providing any dietary advice. The multidomain intervention studies all employed dietary advice in combination with a physical activity component, except for Sheffler et al. [42], which employed motivational strategies to improve adherence. Furthermore, except for the LIILAC study [38], all the multidomain interventions also included a cognitive component.

Designing a proper control group is inherently difficult in dietary studies, and the studies addressed this issue in different ways. In 4 studies, either no behavioral intervention was administered to the control group without any further instructions, or study participants were just encouraged to keep their habitual diet (MAPT [[21], [22], [23]], Johari et al. [34], LIILAC [38], and MedLey [35]). In the NU-AGE [36], FINGER [[24], [25], [26], [27], [28], [29], [30], [31]], and MIND [43] trials, general health or dietary advice was given, and in the BBL-CD [37] and eMIND [39] trials, the control group received online access to educational modules. In all cases, the advice or online resources were less extensive than that received by the intervention group. Instead of using a control group that received dietary advice, the studies by Bayer-Carter et al. [32] and Krikorian et al. [33] compared 2 different types of diets. Finally, Sheffler et al. [42] used a control group that only received nutritional advice, compared to the active arm, which also received strategies to improve motivation and adherence. Table 1 [[21], [22], [23], [24], [25], [26], [27], [28], [29], [30], [31], [32], [33], [34], [35], [36], [37], [38], [39], [40], [41], [42], [43]] presents an overview of all included studies.

**TABLE 1:** Overview of included studies.

Author Country	Study	Design Type of intervention	Size	Selected intervention group	Sex Mean age (y)	Baseline cognition	Mode of intervention	Dietary intervention	Description of intervention	Description of control	Duration	Adherence
Andrieu et al. [21], 2017 France & Monaco	MAPT	Multicenter RCT 4 parallel groups ∗MD + P ∗P ∗MD + PUFAs + P ∗PUFAs	Overall *n* = 1652 MMIT *n* = 1525	MD + P (*n* = 390) vs. P (*n* = 380)	MD + P: 252 F/138 M 75.0 ± 4.1 P: 252 F/128 M 75.1 ± 4.3	∗Mild self-reported memory complaints ∗MMSE > 24	∗Cognitive training ∗Physical activity advice ∗Nutritional advice	Nutritional advice from the French national nutrition and health program.	2 mo/12 group sessions with 15 min dietary advice ∗1st mo 2/wk ∗2nd mo 1/wk Key messages reinforced 1/mo	Placebo supplement without any behavioral intervention	3 y	Adh if attended ≥75% of the sessions
Tabue-Teguo et al. [22], 2018 France & Monaco			Overall *n* = 1652 This study *n* = 1464	MD + P: FRIED = 0 (*n* = 212) FRIED > 0 (*n* = 165) vs. P: FRIED = 0 (*n* = 212) FRIED > 0 (*n* = 160)	Not specified							
Giudici et al. [23], 2020 France & Monaco			Overall *n* = 1652 This study *n* = 1445	MD + P (*n* = 374) vs. P (*n* = 356)	MD + P: 241 F/133 M 75.0 ± 4.1 P: 236 F/120 M 75.1 ± 4.4							
Ngandu et al. [24], 2015 Finland	FINGER	Multicenter RCT 2 parallel groups ∗MD ∗Health advice	Overall *n* = 1260 MMIT *n* = 1190	MD (*n* = 591) vs. Control (*n* = 599)	MD 267 F/324 M 69.5 ± 4.6 Control 284 F/315 M 69.2 ± 4.7	∗CADEI: ≥6 ∗CERAD: mean level or slightly lower than expected for age	∗Dietary counseling ∗Physical exercise program ∗Cognitive training ∗Management of metabolic and vascular risk factors (baseline, 6, 12 & 24 mo)	Dietary advice based on Finnish nutrition recommendations with 9 targets 1. Saturated/ trans fats <10E% 2. PUFAs: 5-10 E% 3. Sucrose: 10 E% 4. Protein 10-20 E% 5. Alcohol: 5 E% 6. Fiber: 3 g/MJ 7. Veggies: 200 g/d 8. Fruit and berries: >200 g/d 9. Fish: 2/wk	∗3 individual counseling sessions ∗6 group sessions	General health advice & feedback about metabolic risk factors at baseline, 6, 12, & 24 mo	2 y	Self-reported Nutrition: 100%
Rosenberg et al. [25], 2018 Finland												NR
Solomon et al. [26], 2018 Finland			*APOE* data *n* = 1109 *APOE* carriers *n* = 362	*APOE* carriers MD (*n* = 173) vs. Control (n = 189)	MD 81 F/92 M 69.21 ± 4.5 Control 94 F/95 M 68.74 ± 4.5							NR
Lehtisalo et al. [27], 2019 Finland			Overall *n* = 1260 Dietary+ cognitive data *n* = 1155	MD (*n* = 571) vs. Control (*n* = 584)	MD 263 F/308 M 69.5 ± 4.6 Control 278 F/306 M 69.1 ± 4.7							Adh score measured by 3-d food records; participants received 1 point for goal achieved (0–9)
Stephen et al. [28], 2019 Finland			Overall *n* = 1260 MRI data *n* = 132	MD (*n* = 68) vs. Control (*n* = 64)	MD 29 F/39 M 70.3 Control 33 F/31 M 69.8							NR
Stephen et al. [29], 2020 Finland			Overall *n* = 1260 DTI data *n* = 60	MD (*n* = 34) vs. Control (*n* = 26)	MD 14 F/34 M 70.02 ± 4.2 Control 13 F/13 M 69.5 ± 3.9							NR
Ngandu et al. [30], 2022 Finland			Overall *n* = 1259	MD Non-adh (*n* = 132) Partial adh (*n* = 265) Adh (*n* = 234) Vs. Control (*n* = 628)	MD 301 F/330 M 69.0 ± 4.7 Non-adh: 57 F/75 M 69.5 ± 5 Partially adh: 124 F/141 M 69.5 ± 4.8 Adh: 105 F/129 M 68.2 ± 4.3 Control 301 F/327 M 68.7 ± 4.7							1.Participation in offered activities 2. Participant life changes Adh to the diet measured through 3-d food records; participants received 1 point for goal achieved (0–9) Adh the diet *n* = 557.88
Neuvonen et al. [31], 2022 Finland			Overall *n* = 1260 Zung score data *n* = 1125	MD Baseline (*n* = 550) 12 mo (*n* = 503) 24 mo (*n* = 482) vs. Control Baseline (*n* = 575) 12 mo (*n* = 527) 24 mo (*n* = 493)	Zung data 502 F/373 M 69.2 ± 4.7							NR
Bayer-Carter et al. [32], 2011 USA	None	RCT 2 parallel groups Dietary intervention ∗HIGH diet ∗LOW diet	Overall *n* = 49 aMCI *n* = 29 Healthy *n* = 20	aMCI HIGH diet (*n* = 15) vs. aMCI LOW diet (*n* = 14)	aMCI HIGH diet: 6 F/9 M 68.1 ± 6.9 aMCI LOW diet: 7 F/7 M 67.1 ± 6.8	∗aMCI: diagnosed by a NPB, when delayed memory scores ≥1.5 SDs	Full diet intervention	HIGH diet: ∗Fat = 45% (saturated fat > 25%) ∗Carbs: 35%-40% (GI >70) ∗Protein: 15%-20% LOW diet: ∗Fat: 25% (saturated fat <7%) ∗Carbs: 55%-60% (GI <55) ∗Protein: 15%-20%	All food was delivered 2/wk to participants home	HIGH vs. LOW	4 wk	Measured by everyday food records. Range of non-adh incidents/wk: 1.23-1.80
Krikorian et al. [33], 2012 USA	None	Randomized intervention study with 2 groups Dietary intervention ∗H. Carb ∗L. Carb	Overall *n* = 23	H. Carb (*n* = 11) vs. L. Carb (*n* = 12)	Overall 13 F/10 M H. Carb 71 ± 8 L. Carb 68 ± 3	∗MCI according to the CDR	High vs. low carbohydrate diet	∗H. Carb: 50% of calories ∗L. Carb: 5%-10% of calories to induce ketosis	Dietary education and counseling at baseline, and weekly contacts throughout the study	H. Carb vs. L. Carb	6 wk	Weekly contacts to promote adh
Johari et al. [34], 2014 Malaysia	None	RCT 2 groups MD health education ∗MD ∗Control	Overall *n* = 35	MD (*n* = 17) vs. Control (*n* = 18)	MD 6 F/11 M 66.9 ± 4.2 Controls 13 F/5 M 63.5 ± 2.9	∗MCI (by Petersen 2004 criteria)	Nutrition and lifestyle education to enhance elderly memory obtained from a booklet based on recent recommendations	Dietary advice according to the booklet: ∗ Eat more fish, foods rich in folic acid, fruits, and vegetables ∗No alcohol	Education sessions based on the booklet 1/mo	Unclear + Placebo capsule 3/d for 12 mo	12 mo	Monitored monthly through individual and group counseling sessions
Knight et al. [35] 2016 Australia	MedLey	RCT 2-cohort parallel group comparison Dietary intervention ∗MedDiet ∗HabDiet	Overall *n* = 166 Analyzed *n* = 137	MedDiet (*n* = 70) vs. HabDiet (*n* = 67)	MedDiet 33 F/37 M 72.1 ± 4.9 HabDiet 41 F/27 M 72.0 ± 5.0	Normal cognitive function DemTect ≥ 13	Dietary instructions from a dietitian on the Australianized Mediterranean-type diet	MedDiet characterized by small amounts of red meat, processed deli meats, and high-sugar foods; moderate amounts of dairy and poultry; and a high plant food content including abundant EVOO	∗Dietary individual counseling at baseline ∗Provided with food items meeting the MedLey guidelines ∗Dietary monitoring every 2 wk	∗Dietary individual monitoring at baseline ∗Encourage to keep habitual Australian diet (HabDiet) ∗Provided monetary gift vouchers to local food supermarkets ∗Dietary monitoring every 2 wks	6 mo	∗MUFA/SFA ratio ∗CRTs in blood ∗Urinary metabolites ∗Daily food check list ∗FFQ ∗3-d WFR Adh of 92%
Marseglia et al. [36], 2018 UK, Poland, France, Italy, NL	NU-AGE	Multicenter RCT with 2 parallel groups Dietary intervention ∗NU-AGE diet ∗Habitual diet	Overall *n* = 1279	NU-AGE diet (*n* = 641) vs. Habitual diet (*n* = 638)	NU-AGE 367 F/271 M 70.7 ± 0.2 Habitual diet 353 F/288 M 71.1 ± 0.2	Free from dementia, passed baseline cognitive test	NU-AGE diet, consisted of individually tailored Mediterranean-like diet advice targeting dietary recommendations for older adults from the 5 included countries	Mediterranean-like diet advice targeting dietary recommendations for older adults from the 5 included countries	∗Individual education 1/mo ∗Motivational counseling at 4 & 8 mo ∗Provided with food items meeting the NU-AGE guidelines	∗Encouraged to follow a habitual diet ∗Received a leaflet with national dietary guidelines.	1 y	Adherence to the NU-AGE diet was measured over follow-up, and categorized into tertiles (low, moderate, high)
McMaster et al. [37], 2020 Australia	BBL-CD	Single blind 2-armed RCT with ∗MD ∗Control	Overall *n* = 119 Analyzed *n* = 96	MD (*n* = 57) vs. Control (*n* = 62)	Intervention 35 F/22 M 72.8 ± 5.3 Control 38 F/24 M 73.3 ± 5.8	∗MCI ∗SCD	Four online educational modules with an 8-wk duration: 1. Dementia and lifestyle risk factors 2.Mediterranean diet 3.Physical activity 4.Cognitive engagement MD cohort had additional active face-to-face sessions to practice all 4 modules	Mediterranean diet	At wk 3, 1-h appointment with dietitian Follow-up meetings at wk 10 & 21	The control condition involved same online 8-wk educational modules	6 mo	Strong adh of the MD to the diet according to the MDAS & ARFS
Hardman et al. [38], 2020 Australia	LIILAC	RCT 2×2 factorial design MD ∗Exercise ∗MedDiet ∗Exercise + diet ∗Control	Overall *n* = 102	MedDiet (*n* = 25) vs. Control (*n* = 27) vs. Exercise+ diet (*n* = 24)	MedDiet 20 F/5 M 77.68 ± 7.38 Control 18 F/9 M 78.22 ± 5.81 Exercise + diet 17 F/7 M 76.54 ± 7.37	∗MMSE >24	1. Exercise 2. MedDiet 3. Exercise + diet 4. Control	Mediterranean-style menu plan	∗No dietitian or food training was given ∗Recommended to follow a 6-wk diet and menu plan including a collection of recipes Mediterranean style including EVOO ∗EVOO was given	Controls maintained habitual lifestyle and diet	6 mo	Daily self-adh score ranging 1-4
de Souto Barreto et al. [39] France	eMIND	RCT 2 parallel groups MD web-based	Overall *n* = 120 Completed *n* = 109	MD (*n* = 54) vs. Control (*n* = 55)	MD 31 F/29 M 2.2 ± 5.7 Control 38 F/22 M 73.2 ± 5.3	∗Subjective memory complaints without dementia ∗MMSE >24	∗Nutrition ∗Exercise ∗Cognitive training	Nutritional advice based on PNNS	MD cohort received a tablet and an accelerometer Access to a multidomain platform Advice every 15 d from new (videos)	Controls only received accelerometers and a link to multidomain activities produced by the research team	6 mo	Adh assessed by clicking on the MD content Adh was considered when clicking ≥75% of content Study reported low compliance
Chatterjee et al. [40], 2022 India	MISCI	RCT 4 parallel groups MD a) Cognitive training b) a + Med diet c) b + exercise d) Control	Overall *n* = 60 Completed *n* = 60	*a) n* = 15 *b) n* = 15 *c) n* = 15 *d) n* = 15	a) 4 F/11 M 68 ± 6.46 b) 7 F/8 M 68.93 ± 5.27 c) 5 F/10 M 65.2 ± 3.73 d) 6 F/9 M 71.07 ± 6.93	∗Subjective cognitive impairment ∗CDR = 0 ∗GDS < 4	∗Cognitive training (computer-based) ∗Med diet ∗Exercise (in-person)	Diet plan based on FINGER trial diet, modified by experts on the Indian counseling of medical research	MED at home, follow-up by the project dietitian by telephone 1/wk and physically at the hospital 1/mo using a food recall sheet	Health awareness instructions (unclear)	6 mo	Authors reported 100% compliance However, how it was performed was not explained
Osuka et al. [41], 2022 Japan	—	Pilot RCT ∗Intervention ∗Control	Overall *n* = 88 Completed *n* = 58	Intervention *n* = 35 vs. Control *n* = 34	Intervention 19 F/16 M 73 Control 16 F/18 M 74	Working >4 d/mo	8 sessions of exercise, nutrition, and psychosocial programs 1/wk	Nutrition advice and food recall	The importance of eating the 10 food groups and the roles of nutrients Record intake score for the 10 food groups at home daily	The control was not provided for any program	8 wk	Adherence evaluated by the withdrawal/ drop-out rates & completion rate of the nutrition diary 3 participants did not report
Sheffler et al. [42], 2023	None	RCT 2 parallel groups ∗Mediterranean ketogenic nutrition adherence (MKNA) ∗Mediterranean ketogenic nutrition education (MKNE)	Overall *n* = 58 Completed *n* = 58	MKNA *n* = 29 vs. MKNE *n* = 29	Overall study population 47 F/11 M 72.91 ± 6.35	Median score of MoCA 25.91; Memory compliant scale 4.24; RBANS A total score 102.05 ± 14.94; delayed memory index 101.60 ± 16.62	7 sessions of nutritional advice	Nutritional advice based on MKN	∗MKNA arm: 7 1-hr sessions for MKN as well as MI-BCT strategies ∗MKNE arm: 7 1-hr sessions for MKN	Control (MKNE) lacked MI-BCT strategies to improve adherence	6 wk	Weekly self-reported adh across 6-wk program ∗MKNA arm 5.97 ± 2.53 ∗MKNE arm 4.49 ± 2.21
Barnes et al. [43], 2023	MIND	RCT 2 parallel groups ∗MIND diet with mild caloric restriction for weight loss ∗Habitual diet with same mild caloric restriction for weight loss	Overall enrolled *n* = 604 *n* = 201 completed 36-mo MRI	MIND diet ∗*n* = 301 randomized ∗*n* = 101 completed 36-mo MRI Control diet ∗*n* = 303 randomized ∗*n* = 100 completed 36-mo MRI	MIND diet 196 F/105 M 70.4 ± 4.2 y Control diet 197 F/106 M 70.4 ± 4.2 y	Global cognition score ∗MIND 0.0 ± 0.6 ∗Control 0.0 ± 0.5	Dietary counseling for all participants via telephone	Dietary counseling for the MIND diet group to incorporate foods from MIND diet and MIND diet recipes	Counseling offered once a week for first 6 mo, every other week second 6 mo, and twice monthly during years 2 and 3	Control group consisted of same frequency of consultation but focused on calorie tracking, portion control, etc.	3 y	Biochemical analyses of antioxidant nutrient levels in blood (lutein, zeaxanthin, and α- and β-carotene) support adherence to MIND diet Weight loss ∗MIND: −5.0 kg ∗Control: −4.8 kg

adh, adherence; aMCI, amnestic mild cognitive impairment; APOE, apolipoprotein E; ARFS, Australian Recommended Food Score; BBL-CD, Body, Brain, Life for Cognitive Decline; CADEI, cardiovascular risk factors; Carbs, carbohydrates; CDR, Clinical Dementia Rating; CERAD, Consortium to Establish a Registry for Alzheimer’s Disease neuropsychological battery; CRT, carotenoid; DemTect, cognitive screening test to support the diagnosis of MCI; E%, total energy intake; EVOO, extra virgin olive oil; F, female; FFQ, food frequency questionnaire; FINGER, Finnish Geriatric Intervention study to Prevent Cognitive Impairment and Disability; FRIED, Fried score for frailty status; GDS, Geriatric Depression Scale; GI, glycemic index; H. Carb, high carbohydrate diet; HabDiet, habitual diet; HIGH, high saturated fat/high glycemic index diet; L. Carb, low carbohydrate diet; LOW, low saturated fat/low glycemic index diet; M, male; MAPT, Multidomain Alzheimer Preventive Trial; MCI, mild cognitive impairment, MD, multidomain intervention; MDAS, Memorial Delirium Assessment Scale; MEDAS, Mediterranean Diet Adherence Screener; MedDiet, Mediterranean diet; MI-BCT, motivation strategies and behavioral change techniques; MIND, Mediterranean-DASH Intervention for Neurodegenerative Delay; MKNA, Mediterranean ketogenic nutrition adherence; MKNE, Mediterranean ketogenic nutrition education; MMIT, modified intention to treat; MMSE, Mini-Mental State Examination; MoCA, Montreal Cognitive Assessment; MRI, magnetic resonance imaging; MUFA, monosaturated fatty acid; NPB, neuropsychological battery; NR, not reported; P, placebo; PNNS, Programme National Nutrition Sant; PUFA, polyunsaturated fatty acid; RBANS, Repeatable Battery for the Assessment of Neurophysiological Status; RCT, randomized controlled trial; SCD, subjective cognitive decline; SD, standard deviation; SFA, saturated fatty acid; WFR, weighted food record.

### Cognitive task-based outcomes related to memory

#### Working memory

Several studies used the Trail Making Test B to assess working memory, and none of the studies observed significant effects of diet on performance in this test [21,22,[32], [33], [34], [35], [36], [37],[39], [40], [41], [42]] (Table 2). In addition, several other tests to assess working memory were employed in the included studies. Johari et al. [34] did not find significant differences in the Digit Span test. In the whole-diet intervention trial conducted by Bayer-Carter et al. [32], the intervention had no effect on immediate story recall or list recall.

**TABLE 2:** Studies investigating effects of whole-diet interventions on memory-related cognitive tasks

Cognitive function	Task	#Studies with positive significant effect	#Studies with no positive significant effect
Working memory	Trail making test part B (Krikorian et al. [33], 2012^1^; McMaster et al. [37], 2020^2^; Johari et al. [34], 2014^2^; Bayer-Carter et al. [32], 2011^1^; Tabue-Teguo et al. [22], 2018^2^; Andrieu et al. [21], 2017^2^; Osuka et al. [41], 2022^2^) Digit Span (Johari et al. [34], 2014^2^) Immediate story recall (Bayer-Carter et al. [32], 2011^1^) List recall (Bayer-Carter et al. [32], 2011^1^)		Krikorian et al. [33], 2012^1^; McMaster et al. [37], 2020^2^; Johari et al. [34], 2014^2^; Bayer-Carter et al. [32], 2011^1^; Tabue-Teguo et al. [22], 2018^2^; Andrieu et al. [21], 2017^2^; Osuka et al. [41], 2022^2^ Johari et al. [34], 2014^2^ Bayer-Carter et al. [32], 2011^1^ Bayer-Carter et al. [32], 2011^1^
Visuospatial memory	Visual reproduction I and II (Johari et al. [34], 2014^2^) Brief visuospatial memory test (BVMT) (Bayer-Carter et al. [32], 2011^1^) Visual-spatial composite score (Knight et al. [35], 2016^1^)		Johari et al. [34], 2014^2^ Bayer-Carter et al. [32], 2011^1^ Knight et al. [35], 2016^1^
Episodic memory	Verbal paired associate learning test (V-PAL) (Krikorian et al. [33], 2012^1^) Delayed story recall (Bayer-Carter et al. [32], 2011^1^) Delayed list recall (Bayer-Carter et al. [32], 2011^1^) Delayed visual memory recall (Bayer-Carter et al. [32], 2011^1^) Free and cued selective reminding test (FCSRT) (de Souto Barreto et al. [39], 2021^2^; Tabue-Teguo et al. [22], 2018^2^; Andrieu et al. [21], 2017^2^) Episodic memory domain [computed from mean of Z-scores from sum of immediate word list memory (WLM) and Babcock story recall test (BSRT) and delayed WLM and BSRT tasks] (Marseglia et al. [36], 2018^1^)	Krikorian et al. [33], 2012^1^ Bayer-Carter et al. [32], 2011^1^ Marseglia et al. [36], 2018^1^	Bayer-Carter et al. [32], 2011^1^ Bayer-Carter et al. [32], 2011^1^ de Souto Barreto et al. [39], 2021; Tabue-Teguo et al. [22], 2018^2^; Andrieu et al. [21], 2017^2^
Subjective memory assessment	Visual analog scales measuring memory functioning and consequences in everyday life (Andrieu et al. [21], 2017^2^)		Andrieu et al. [21], 2017^2^
Delayed memory	Repeatable battery for the assessment of neuropsychological status – delayed memory Index (Sheffler et al. [42], 2023^2^)	Sheffler et al. [42], 2023^2^	
Composite memory scores	Memory composite score (Knight et al. [35], 2016^1^) Post Graduate Institute Memory Score (PGI-MS) (Chatterjee et al. [40], 2022^2^)	Chatterjee et al. [40], 2022^2^	Knight et al. [35], 2016^1^

1 Indicates interventions that investigated the effects of a whole-diet intervention alone.

2 Indicates studies that investigated effects of diet as a component of a multidomain intervention.

#### Visuospatial memory

Johari et al. [34] assessed visuospatial memory using the Visual Reproduction I and II tests and observed no difference between those individuals receiving nutritional and lifestyle education and the control group. The whole-diet intervention in which food was delivered to participants did not significantly impact performance in the Brief Visuospatial Memory Test [32]. Furthermore, the 6-mo MedLey intervention found no differences between groups on a visual-spatial memory composite score (computed as the *z*-score of the Benton Visual Retention Test).

#### Episodic memory

Long-term memory performance was assessed using the Verbal Paired Associate Learning Test (V-PAL) in the intervention with the 6-week high or very low carbohydrate intervention conducted by Krikorian et al. [33]. They found that performance on the V-PAL did not change in the high carbohydrate group but significantly improved in the group receiving the low carbohydrate diet (*P* = 0.01). Moreover, Bayer-Carter et al. [32] found that a low saturated fat/low glycemic index (LOW) diet improved performance in the visual recall in healthy participants and those with MCI (time × diet interaction effect, *P* = 0.04).

In addition, the eMIND intervention investigated the feasibility and acceptability of a 6-mo web-based multidomain intervention consisting of nutritional advice, personalized exercise training, and cognitive training compared to a control group [39]. They measured the effects of the intervention on total recall in the Free and Cued Selective Reminding test (FCSRT) and observed no intervention effects. Similarly, the MAPT trial also found no effects of their multidomain intervention on performance on the FCSRT [21,22].

In the NU-AGE trial, participants were randomized into a group receiving individually tailored dietary advice (NU-AGE diet) or a group that followed a habitual diet [36]. At 1 y, there was no difference between groups in an episodic memory domain. However, participants with higher adherence to the NU-AGE diet showed significant improvements in episodic memory (*P* = 0.025).

#### Subjective memory assessment

In the multidomain MAPT trial, the participants self-assessed memory functioning in everyday life using analog scales [21]. There was no difference when comparing any of the 3 intervention arms to the placebo in this measurement of subjective memory.

#### Delayed memory

Sheffler et al. [42] conducted a 6-wk intervention to compare the effects of Mediterranean ketogenic nutrition (MKN) education and MKN education with motivation interviewing strategies and behavior change techniques, with feasibility, acceptability, and adherence as primary outcomes. Secondary outcomes included the Delayed Memory Index of the Repeatable Battery for the Assessment of Neuropsychological Status (RBANS). There was statistically significant improvement across both arms from baseline to 6 wk (*P* = 0.042). However, there were no differences between groups, and there were no differences in scores from baseline to the 3-mo postintervention follow-up visit.

#### Composite memory scores

The 6-month intervention by Knight et al. [35], comparing the effects of a Mediterranean dietary pattern and the habitual diet on memory, found no differences between groups on a memory composite score (computed as mean of *z*-scores on Rey and Schmidt’s Rey Auditory Verbal Learning Test, Digit Span Forward, Digit Span Backward, and the Letter Number Sequencing tests). In addition, Chatterjee et al. [40] conducted a 24-wk multidomain intervention in comparison with health awareness among older adults with subjective cognitive impairment (MISCI-Trial). The multidomain intervention resulted in improvement in the Post Graduate Institute Memory Scale total score (*P* = 0.001), compared to a control group.

### Other cognitive task-based outcomes

#### Global cognition

Several studies used the MMSE to assess global cognition, and none found a significant effect [[21], [22], [23], [24],^32^,[34], [35], [36], [37], [38], [39], [40], [41], [42], [43]] (Table 3). Furthermore, Johari et al. [34] found no significant effects of the intervention on 2 other assessments of global cognition—the Matrix Reasoning and Clock Drawing tests.

**TABLE 3:** Studies investigating effects of whole-diet interventions on other cognitive tasks.

Cognitive function	Task	#Studies with positive significant effect	#Studies with no positive significant effect
Global cognition	Mini-Mental State Examination (MMSE) (Johari et al. [34], 2014^1^; de Souto Barreto et al. [39], 2021^1^; Tabue-Teguo et al. [22], 2018^1^; Andrieu et al. [21], 2017^1^; Marseglia et al. [36], 2018^2^) Matrix reasoning (Johari et al. [34], 2014^1^) Clock drawing test (Johari et al. [34], 2014^1^)		Johari et al. [34], 2014^1^; de Souto Barreto et al. [39], 2021^1^; Tabue-Teguo et al. [22], 2018^1^; Andrieu et al. [21], 2017^1^; Marseglia et al. [36], 2018^2^ Johari et al. [34], 2014^1^ Johari et al. [34], 2014^1^
Processing speed	Speed of processing composite score (Knight et al. [35], 2016^2^) Perceptual speed domain (Marseglia et al. [36], 2018^2^) Digit symbol (Johari et al. [34], 2014^1^; McMaster et al. [37], 2020^1^; de Souto Barreto et al. [39], 2021^1^; Tabue-Teguo et al. [22], 2018^1^; Andrieu et al. [21], 2017^1^) Trail making test part A (Bayer-Carter et al. [32], 2011^2^; Tabue-Teguo et al. [22], 2018^1^; Andrieu et al. [21], 2017^1^)	McMaster et al. [37], 2020^1^	Knight et al. [35], 2016^2^ Marseglia et al. [36], 2018^2^ Johari et al. [34], 2014^1^; de Souto Barreto et al. [39], 2021^1^; Tabue-Teguo et al. [22], 2018^1^; Andrieu et al. [21], 2017^1^ Bayer-Carter et al. [32], 2011^2^; Tabue-Teguo et al. [22], 2018^1^; Andrieu et al. [21], 2017^1^
Language	Verbal abilities domain (Marseglia et al. [36], 2018^2^)		Marseglia et al. [36], 2018^2^
Executive function	Block design test (Johari et al. [34], 2014^1^) Category fluency for vegetables (McMaster et al. [37], 2020^1^) Stroop test/interference condition (Bayer-Carter et al. [32], 2011^2^) Stroop color word test (Chatterjee et al. [40], 2022^1^) Verbal fluency test (Bayer-Carter et al. [32], 2011^2^) Category naming test (CNT) (de Souto Barreto et al. [39], 2021^1^; Tabue-Teguo et al. [22], 2018^1^; Andrieu et al. [21], 2017^1^) Controlled oral word association test (COWAT) (de Souto Barreto et al. [39], 2021^1^; Tabue-Teguo et al. [22], 2018^1^; Andrieu et al. [21], 2017^1^) Executive function composite score (Knight et al. [35], 2016^2^) Executive function domain (Marseglia et al. [36], 2018^2^)	Johari et al. [34], 2014^1^	McMaster et al. [37], 2020^1^ Bayer-Carter et al. [32], 2011^2^ Chatterjee et al. [40], 2022^1^ Bayer-Carter et al. [32], 2011^2^ de Souto Barreto et al. [39], 2021^1^; Tabue-Teguo et al. [22], 2018^1^; Andrieu et al. [21], 2017^1^ de Souto Barreto et al. [39], 2021^1^; Tabue-Teguo et al. [22], 2018^1^; Andrieu et al. [21], 2017^1^ Knight et al. [35], 2016^2^ Marseglia et al. [36], 2018^2^
Motor control	Stroop test/matching condition (Bayer-Carter et al. [32], 2011^2^) Constructional praxis (CERAD copy task) (Marseglia et al. [36], 2018^2^)		Bayer-Carter et al. [32], 2011^2^ Marseglia et al. [36], 2018^2^
Visuospatial ability	3D cube copy test (Osuka et al. [41], 2022^1^)		Osuka et al. [41], 2022^1^
Composite measurements	Swinburne University Computerized Cognitive Assessment Battery (SUCCAB) (Hardman et al. [38], 2020^1^) Neuropsychological test battery (NTB) z-score (Ngandu et al. [24], 2015^1^) Alzheimer’s Disease Assessment Scale – Cognitive 11 (ADAS-Cog 11) (McMaster et al. [37], 2020^1^) Pfeffer Functional Activities Questionnaire (PFAQ) (McMaster et al. [37], 2020^1^) Composite *z*-score (based on mean and SD of ADAS-Cog, PFAQ, TMT-B, and SDMT) (McMaster et al. [37], 2020^1^) Composite score combining the Z-scores of 4 scales (MMSE; DSST, WAIS-R; FCSRT; CNT) (de Souto Barreto et al. [39], 2021^1^) Composite Z-score (combining free and total recall of free and cued selective reminding test, 10 MMSE orientation items, digit symbol substitution test score from Wechsler Adult Intelligence Scale – Revised, and category naming test) (Andrieu et al. [21], 2017^1^) Intrinsic capacity (IC) Z-score (Giudici et al. [23], 2020^1^) Total age-related cognitive function score (composite of 11 tests) (Knight et al. [35], 2016^2^) Global cognition [measured with MMSE and Consortium to Establish a Registry for Alzheimer’s Disease (CERAD)-total score] (Marseglia et al. [36], 2018^2^) Repeatable Battery for the Assessment of Neuropsychological Status (Sheffler et al. [42], 2023^1^) Global composite score (compiled from z-scores averaged across 12 publicly available cognitive function tests) (Barnes et al. [43], 2023^2^)	Ngandu et al. [24], 2015^1^ McMaster et al. [37], 2020^1^ Marseglia et al. [36], 2018^2^ Sheffler et al. [42], 2023^1^	Hardman et al. [38], 2020^1^ McMaster et al. [37], 2020^1^ McMaster et al. [37], 2020^1^ de Souto Barreto et al. [39], 2021^1^ Andrieu et al. [21], 2017^1^ Giudici et al. [23], 2020^1^ Knight et al. [35], 2016^2^ Barnes et al. [43], 2023^2^

Abbreviations: ADAS-Cog, Alzheimer’s Disease Assessment Scale-Cognitive 11; SDMT, Symbol Digit Modalities Test; WAIS-R, Wechsler Adult Intelligence Scale-Revised.

1 Indicates studies that investigated effects of diet as a component of a multidomain intervention.

2 Indicates interventions that investigated the effects of a whole-diet intervention alone.

#### Processing speed

The BBL-CD intervention showed a significant group × time point interaction (*P* = 0.040) for the Symbol Digit Modalities Test (SDMT), although there were no between-group differences at the 3- or 6-mo follow-up [37]. Johari et al. [34], the eMIND trial [39], and the MAPT trial [21,22] found no effect of multidomain interventions on this task.

#### Language

In addition to assessing perceptual speed, Marseglia et al. [36] constructed a verbal ability composite score. The NU-AGE diet did not significantly affect verbal abilities in this task compared to the habitual diet.

#### Executive function

In the trial conducted by Johari et al. [34], there was a significant improvement in the Block Design Test score in the intervention group compared to the control group (*P* = 0.050). However, Bayer-Carter et al. [32] found no difference in the Stroop Test/Interference condition or the Verbal Fluency Test. No differences were found for 2 measurements of executive function (Category Naming Test and Controlled Oral Word Association Test) in the web-based multidomain 6-mo intervention conducted by de Souto Barreto et al. [39] or in the MAPT trial [21,22].

#### Motor control

Bayer-Carter et al. [32] found no differences between the HIGH and LOW diets in the Stroop Test/matching condition. In the NU-AGE trial [36], there were no differences in the NU-AGE diet versus a habitual diet in the constructional praxis [Consortium to Establish Registry for Alzheimer’s Disease (CERAD) copy task] after the 1-y intervention.

#### Visuospatial ability

The 8-wk multidomain intervention consisting of exercise, nutrition, and psychosocial programs failed to significantly affect performance on the 3-dimensional cubic test in the intervention group compared to the control [41].

#### Composite measurements

A 6-mo 4-armed intervention compared the effects of *1*) an exercise regimen, *2*) adherence to a MedDiet, *3*) combined exercise and diet intervention, and *4*) maintenance of the participant’s current lifestyle on cognition as assessed by the Swinburne University Cognitive Assessment Battery [38]. There were no significant differences between groups for the primary composite score. However, the combined exercise and diet intervention group exhibited a significant improvement in the spatial working memory component compared to controls (*P* < 0.05).

The FINGER trial, which assessed the efficacy of a 2-y multidomain intervention to improve cognition as assessed by a neuropsychological test battery (NTB) compared to a control group, found a significant between-group difference in the change of NTB total score per year (*P* = 0.030) [24]. Moreover, a healthier baseline diet predicted improvement in global cognition during the 2-y FINGER intervention for both groups, associated with improvements in executive function, particularly in the intervention group [27].

An 8-wk trial examined the efficacy of a multidomain intervention to reduce lifestyle risk factors for Alzheimer’s disease and improve cognition in individuals with subjective cognitive decline or MCI [37]. The intervention group had significantly higher composite *z*-scores (a composite score including 4 tests: Alzheimer’s Disease Assessment Scale-Cognitive 11, Pfeffer Functional Activities Questionnaire, Trail Making Test B, and SDMT) at both the 3-mo (*P* = 0.010) and 4-mo (*P* = 0.021) follow-ups.

In the NU-AGE trial, the global cognition score (comprising the MMSE and the CERAD test) was not significantly different between groups [36]. However, participants with higher adherence to the NU-AGE diet showed significant improvements in global cognition (*P* = 0.046).

In the 6-wk intervention examining the effect of motivational strategies to improve adherence to a Mediterranean ketogenic nutrition program, there was statistically significant improvement across both arms from baseline to 6 wk for the RBANS total score (*P* = 0.047) [42]. However, there were no differences between groups, and there were no differences in scores from baseline to the 3-mo postintervention follow-up visit.

Finally, the MIND trial assessed the cognitive effects of the MIND diet with mild caloric restriction compared to a control diet with mild caloric restriction for 3 y [43]. The primary outcome of the study was the change in performance on a battery of 12 publicly available cognitive function tests from baseline to year 3. There was no difference between groups for the global cognition score or for 4 individual cognitive domain scores (episodic memory, executive function, perceptual speed, and semantic memory).

### Additional outcomes related to cognitive function or disease risk

Apart from the outcomes addressed above, several studies assessed additional outcomes related to cognition*.* For example, magnetic resonance imaging (MRI) and positron emission tomography (PET) were used to evaluate structural changes in the brain and amyloid deposition, respectively. Alzheimer’s disease risk biomarkers were measured in cerebrospinal fluid and assessed by genotyping [apolipoprotein E (*APOE)* ε4 variant]. In addition, clinical rating scales and self-reporting instruments were used to assess dementia and Alzheimer’s disease risk and to estimate engagement in leisure time cognitive activities (e.g., crosswords, cultural outings, and social activities), as further described below.

Concerning self-reported outcomes, the eMIND trial found no effect on leisure time cognitive activities from their multidomain intervention [39], whereas the FINGER trial significantly improved a multi-lifestyle score including self-reported frequency of cognitive and social activities [30]. In this trial, a higher multi-lifestyle score was also predictive of improvements in global cognition and executive function. Notably, both Ngandu et al. [30] and Lehtisalo et al. [27] showed that more engagement in cognitive activities at baseline positively impacts postinterventional cognitive outcomes. Moreover, lifestyle risk factors for Alzheimer’s disease were significantly reduced by the multidomain intervention of McMaster et al. [37]. However, the authors suggest that the risk reduction was driven by improvements in protective factors, such as cognitive engagement, rather than change in actual risk factors.

Assessment of brain structure by MRI [28,43] and diffusion tensor imaging [26] showed no postintervention improvements with 1 exception; diffusion tensor imaging detected a decrease in fractional anisotropy in widespread white matter tracts [29], an observation opposite to the authors expectations. Interestingly, in contrast to its pathologic indication, the fractional anisotropy decrease in the intervention group was associated with significant cognitive benefits [29]. In line with Lehtisalo et al. [27] and Ngandu et al. [30], Stephen et al. [29] recognized that baseline characteristics may impact postinterventional cognitive outcomes. For example, they found that the interventional effects on cognitive processing speed were more pronounced in subjects with higher baseline cortical thickness in the entorhinal, inferior and middle temporal, and fusiform regions [28]. In addition, Andrieu et al. [21] showed that presence of amyloid deposition (assessed by amyloid PET) was associated with less cognitive decline (measured as composite *z*-score of 4 different cognitive tests). The authors speculated that this finding may indicate that multidomain intervention may slow cognitive decline especially in people at risk, which is supported by a similar correlation in individuals with high dementia risk [21].

Concerning the biomarkers assessed, Bayer-Carter et al. [32] investigated the effect of 2 different diets on Alzheimer’s disease risk modulation via cerebrospinal fluid and observed changes in APOE and F2-isoprostane concentrations; Aβ40, Aβ42, tau protein, and phospho-tau, however, remained unaffected. Also, the *APOE* ε4 genotype was assessed in several studies and reported both as a characteristic [29,30,35] and relative to interventional effects [21,26,32]. On this note, the multidomain intervention given in the FINGER trial resulted in improved cognition and memory when considering only *APOE* ε4 carriers [26]. In the study by Andrieu et al. [21], however, the *APOE* ε4 genotype did not affect any of the assessed outcomes, as supported by Bayer-Carter et al. [32], who performed a sole dietary intervention. Moreover, neither diet alone nor a combined intervention of diet and exercise affected brain-derived neurotrophic factor levels in blood, as found by Hardman et al. [38].

### Assessment of study quality

**FIGURE 2:**
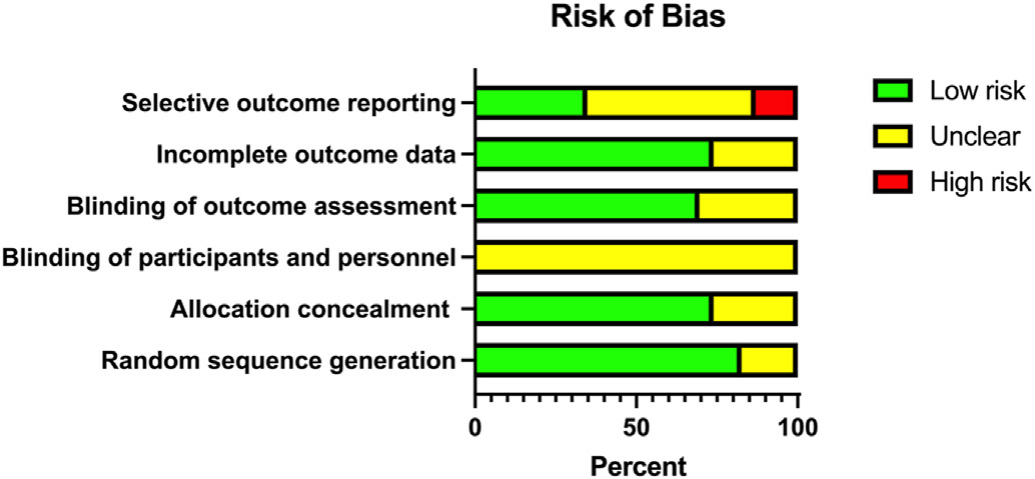
Risk of bias assessment including all 23 articles.

The general estimation of risk of bias in the included studies was Unclear; most of the assessed studies were scored as Unclear on multiple items and all had ≥1 item scored as Unclear. In particular, all studies were scored Unclear on the blinding of participants and personnel (Figure 2). Three studies reported an open-label design [34,37,38], 1 study deviated from its original protocol stating that the study was single-blinded, although the protocol stated open-labeled and unmasked [36]. Another study was rigorously blinded and controlled concerning some parts, whereas other parts (i.e., the multidomain intervention) were unblinded [21,22,23]. As mentioned earlier, we recognize that it is difficult to blind dietary interventions and further consider it unlikely that outcome measures were unduly influenced by lack of intervention blinding in the assessed studies. Hence, we saw reason to score an Unclear risk of bias, rather than a High risk, on this item. Random sequence generation was clear and raised no concern for bias in most studies, but allocation concealment was seldom addressed in sufficient detail. We raise concerns about High risk of bias due to selective outcome reporting in 3 studies [22,23,26], 2 reporting on nonpredefined secondary analysis of data generated from the MAPT and 1 from the FINGER trial.

## Discussion

This systematic review reports on scientific studies investigating the effects of whole-diet interventions on memory and cognition in older adults. To our knowledge, this is the first systematic review to focus a comprehensive and systematic database search on memory outcomes in healthy older adults. Interestingly, only a few of the included studies investigated dietary strategies alone [32,33,35,36,43]. Strikingly, only 4 of these studies alongside 2 multidomain interventions [40,42] reported significant effects on memory function, whereas studies using multidomain approaches seemed to affect other cognitive outcomes. Five multidomain studies showed significant effects on cognition from interventions combining diet with “lifestyle education” [34], motivational strategies [42], or with cognitive training and physical activity [22,24,37]. In addition to its positive effects on episodic memory, one of the studies intervening solely with diet [36] also reported significant effects on global cognition. Notably, this study, the NU-AGE trial, is one of the largest, both in terms of participants included and study duration.

In addition to the task-related outcomes presented, the interventions significantly impacted several other outcomes that are of potential importance to cognition and nervous system function. To list them briefly, multidomain interventions including a whole-dietary intervention component may reduce risk factors for Alzheimer’s disease [37] and potentially influence lifestyle factors, including self-reported frequency of cognitive and social activities [25]. Also, 1 study found a postinterventional decrease in white matter fractional anisotropy being associated with significant cognitive benefits [29], whereas another study showed improvement in cognition and memory in a subgroup analysis of *APOE* ε4 carriers [26]. As in the case of *APOE* ε4 carriers, other baseline characteristics, such as more engagement in leisure time cognitive activities [27,30] and increased cortical thickness [28], seemed to affect the assessed outcomes. A potential implication from these findings is that early intervention may be key to achieve positive effects from dietary/lifestyle interventions, identifying at-risk groups that are more likely to benefit from these interventions.

### Potential mechanisms underlying the effects of whole-diet interventions on memory

Although the positive effects of whole-diet interventions on memory and cognition were limited to a small number of studies, it is important to investigate the mechanisms underlying the effects of these interventions. As mentioned previously, only 4 of the 11 studies addressed the effects of diet alone on cognition [32,33,35,36], whereas diet was a component of a multidomain intervention in the other 7 studies. Based on these articles, it is difficult to ascertain whether a dietary intervention alone is sufficient to affect memory and/or cognition or if a dietary intervention alongside other lifestyle interventions is required. Several reviews have concluded that multidomain interventions have more pronounced effects on cognition than single interventions in healthy older adults, many focusing on the combined effect of exercise and cognitive training [[44], [45], [46]]. A systematic review conducted by Salzman et al. [47] suggests that studies with multidomain interventions are more strongly associated with positive effects on global cognition, memory, executive function, and verbal fluency in older adults with MCI than a single intervention. This may indicate that the interplay between multidomain lifestyle interventions is necessary, and potentially more efficacious, to improve memory and cognition in older adults. To further address this issue, studies with multiple arms for each single intervention and combinations of interventions should be performed, similar to MAPT [[21], [22], [23]], LIILAC [38], and MISCI [40]. However, such studies would require significant financing and large numbers of participants and would be difficult to coordinate.

In addition, it is also necessary to consider whether the interplay between multiple nutrients or if intervening on specific nutrients would be more efficacious. Although the included studies utilized a variety of whole-diet interventions, the majority focused on adherence to the MedDiet, MIND, or other MedDiet-like diets, altering the percentage of dietary fat or carbs consumed, or other dietary advice. The MedDiet consists of increased consumption of olive oil, legumes, whole grains, fruits, vegetables, nuts, and poultry and involves reduced consumption of red meat and refined grains and sugars [48,49]. Unlike the MedDiet, the MIND diet has been specifically designed to improve brain health and prevent dementia and is a combination of the MedDiet and DASH diet [14,50]. Although the MedDiet and MIND diets have been associated with improved cognitive function [3,5], several of the individual components, including nuts [51], olive oil [52], and berries [53], have separately been shown to positively affect memory function and cognition in older adults. Importantly, multi-armed designs could help to separate the diet-related effects from other lifestyle interventions, providing additional insights into the mechanism of action.

There are several mechanisms by which diet could potentially affect brain function. First, adhering to a healthier diet, such as the MedDiet, has been shown to reduce cardiovascular risk factors [54,55], which have been associated with cognitive decline and dementia. Furthermore, improved diet may affect memory and cognitive function by reducing oxidative stress and chronic low-grade inflammation [56]. High levels of high sensitivity C-reactive protein and IL-6 have been correlated with poor cognitive performance in the elderly [57,58]. Furthermore, blood levels of C-reactive protein and IL-6 have also been shown to predict future cognitive decline [59,60]. There is substantial evidence to suggest that many foods and nutrients modulate inflammation both acutely and chronically [[61], [62], [63], [64]]. In addition, changes in diet may influence brain function via modulation of the gut microbiota. An increasing number of studies suggest that communication along the microbiota-gut-brain axis is important for brain development, cognition, and mood regulation and that dysregulation of the gut-brain axis is linked to neurodegenerative and neuropsychiatric disorders [65,66]. Supporting the potential of modulating gut microbiota to impact cognitive function in older adults, a 12-wk probiotic intervention improved mental flexibility, as well as increased serum levels of brain-derived neurotrophic factor [67]. Moreover, the NU-AGE trial resulted in alterations in the gut microbiota that were positively associated with both improved cognitive function and markers of lower frailty and negatively correlated with several inflammatory markers [68]. Further investigation of the potential effects of dietary interventions on memory and cognition in older adults will aid in the design and effectiveness of such interventions.

### Status of the research field and methodological considerations

The findings from several cross-sectional and prospective studies suggest that diet affects health and brain function in older adults [[10], [11], [12]]. According to the findings of this review, such claims may be premature, at least regarding improvements in the cognitive health status of older adults. Whole-diet interventions exploring these outcomes are quite new, with the first one being published in 2011 [32], and several of the other articles were published in the last 5 y. Importantly, only a few randomized trials to date actually “isolate” the effect of diet, as most studies adopt different multidomain interventions. Furthermore, many of the included studies originate from the same trials. For example, the FINGER trial is the basis of 8 of the reviewed articles, whereas the MAPT parented 3. This is somewhat reflected in the risk of bias assessment conducted, as some of the studies report on nonpredefined secondary analysis of data generated from these trials of origin. The risk assessment also serves to identify the blinding problem in lifestyle-focused trials, where all studies were scored as Unclear risk of bias. Not all studies explicitly state that the design is unblinded, which it preferably should be whenever this is the case. Other studies seem to have tried to conceal the group allocation, albeit recognizing it as problematic and practically difficult.

Another design issue prevalent in this research field is underpowering. Many of the included studies did not have memory as primary outcome and were thus not adequately powered to evaluate memory-related outcomes. In the RCTs reviewed here, significant effects were only seen in a limited number of instruments/tasks and most often in subscales, parts of an instrument, or in composite scores. This complicates accurate power estimations in future studies, as there are few positive findings with enough rigor to rely on for this purpose. Hence, it seems this field needs to further mature to provide truly helpful templates.

Furthermore, dietary advice is commonly the implementation method for dietary interventions, as it is the most feasible; however, it is not necessarily the most effective. Based on the findings of our systematic review, we suggest that it may be reasonable to invest resources to advance the field by smaller “proof-of-concept” trials implementing strict interventional administration, for example, by supplying preprepared meals or groceries with accompanying recipes for preparation at home.

Finally, a prevalent issue in this field is the heterogeneity of study design and outcomes selected. The duration of the included studies ranged from as short as 4 wk [32] to 3 y [21,22,23,43]. The 4-wk study conducted by Bayer-Carter et al. [32] showed positive effects on episodic memory, whereas the 3-y MIND trial showed no differences between the MIND and control diets [43]. Therefore, a longer study duration does not necessarily lead to positive effects on memory and cognition. In addition to study duration, the included studies employed a wide variety of cognitive tests, questionnaires, and brain imaging measurements. To better understand the effects of whole-diet intervention on memory, standardization of the outcomes assessed is crucial to compare studies. Finally, several of the studies were conducted in older adults without MCI, dementia risk, or memory complaints. To see more positive effects of whole-diet interventions on memory and cognition, it may be prudent to focus on populations at risk for cognitive decline or those with poor nutritional status. Otherwise, it would potentially be very difficult to observe improvement in cognitively healthy individuals.

### Future directions

To confirm the findings of cross-sectional studies and claims of popular science communication within the field of nutrition, cognition, and aging, sufficiently powered studies with memory as the primary outcome are necessary. In this context, it is noteworthy that most of the current studies are based on multidomain interventions, thereby making it difficult to determine the contribution of diet to the observed effects. Hence, to separate the effects of different lifestyle interventions, the field needs studies “isolating” interventional approaches. This could potentially be accomplished by focusing solely on dietary interventions in parallel groups or by trials with multiple arms but intervening with one single approach in each of them. As there are currently no studies in which participants and study staff are strictly unaware of the different group allocation, developing proper, standardized procedures for blinding is crucial in future studies. Moreover, considering the influence of certain baseline characteristics in producing positive effects [21,26,27,29,30], it may be interesting for future studies to invest resources in thoroughly characterizing the study population to better define groups of “responders” and “nonresponders” in dietary interventions. This may be a way to facilitate future precision nutrition strategies for individually tailored dietary advice to maintain or improve memory among older adults.

## Author contributions

The authors’ responsibilities were as follows – LT, CB, JR, MFRR, JP, AH: defined the search string; LT, CB, JR, MFRR, LBJ, LS, AH: title screening; LT, CB, JR, MFRR, LBJ, LS, AH: abstract screening; LT, CB, JR, MFRR, AH: data extraction; LT: risk of bias assessment for all included articles; LT, CB, JR, MFRR, LBJ, LS, AH : writing the original draft, reviewing, and editing; AH, LT: led the writing process and finalized the manuscript; and all authors: read and approved the final manuscript.

## Conflict of interest

The authors report no conflicts of interest.

## Data Availability

Data described in the manuscript, code book, and analytic code will be made available upon request.
