# Supplementary material for: The Effect of Whole-Diet Interventions on Memory and Cognitive Function in Healthy Older Adults – A Systematic Review
Source: Adv Nutr. 2024 Aug 19;15(9):100291. doi: 10.1016/j.advnut.2024.100291 (PMC11405642; doi:10.1016/j.advnut.2024.100291)
Supplement: Multimedia component 1 [file mmc1.docx]

**Supplementary Material S1: Detailed Search Description**

Medline (Database(s): **Ovid MEDLINE(R) ALL**1946 to May 03, 2024)

| Search terms | | | Total number of results |
| --- | --- | --- | --- |
| Elderly | | | |
|  | 1 | exp Aged/ |  |
|  | 2 | exp Geriatrics/ |  |
|  | 3 | Cognitive Aging/ |  |
|  | 4 | Geriatric Nursing/ |  |
|  | 5 | (elder* or eldest or frail* or geriatri* or "old age" or "older adult*" or "older age" or "older female*" or "older male*" or "older man" or "older men" or "older patient*" or "older people*" or "older person*" or "older population" or "older subject*" or "older woman" or "older women" or "oldest old" or senior* or senium or "very old").ab,kf,ti. |  |
|  | 6 | 1 or 2 or 3 or 4 or 5 |  |
| Diet intervention | | | |
|  | 7 | exp Diet/ |  |
|  | 8 | exp Diet Therapy/ |  |
|  | 9 | dh.fs. |  |
|  | 10 | exp Feeding Behavior/ |  |
|  | 11 | (diet* or atherogenic or "carbohydrate restricted" or "low carbohydrate" or "high protein low carb*" or "carbohydrate restricted high protein" or "south beach" or atkins or ketogenic or keto or "fat restricted" or "low fat" or "fat free" or "gluten free" or "high fat" or "high protein" or mediterranean or paleolithic or "stone age" or paleo or caveman or "hunter gatherer" or "protein restricted" or "low protein" or "protein free" or "sodium restricted" or "low sodium" or "low salt" or "salt free" or vegetarian* or "plant based" or macrobiotic or vegan*).ab,kf,ti. |  |
|  | 12 | ((food* or nutrit* or eat*) adj2 (pattern* or habit* or intervent* or therap*)).ab,kf,ti. |  |
|  | 13 | 7 or 8 or 9 or 10 or 11 or 12 |  |
| Working memory/short term memory | | | |
|  | 14 | Memory/ |  |
|  | 15 | Memory, Short-Term/ |  |
|  | 16 | Executive Function/ |  |
|  | 17 | Neuropsychological Tests/ |  |
|  | 18 | exp "Memory and Learning Tests"/ |  |
|  | 19 | Wisconsin Card Sorting Test/ |  |
|  | 20 | memory.ab,kf,ti. |  |
|  | 21 | executive function.ab,kf,ti. |  |
|  | 22 | ("embedded figures test" or "figural fluency test" or "Rey Osterrieth complex figure test" or "rod and frame test" or "Simon task" or "Stroop test" or "tower of London test" or "trail making test" or "Wisconsin Card Sorting Test" or "process dissociation procedure" or "Buschke selective reminding test" or "California verbal learning test" or "Hopkins verbal learning test" or "Rey auditory verbal learning test" or "word list recall").ab,kf,ti. |  |
|  | 23 | ("immediate recall*" or "cued recall" or "serial recall").ab,kf,ti. |  |
|  | 24 | 14 or 15 or 16 or 17 or 18 or 19 or 20 or 21 or 22 or 23 |  |
| Combined sets | | | |
|  | 25 | 6 and 13 and 24 |  |
| Limits: 2002-, english language | | | |
|  | 26 | limit 25 to (english language and yr="2002 - 2024") | 1409 |

Embase.com (Elsevier)

| Search terms | | | Total number of results |
| --- | --- | --- | --- |
| Elderly | | | |
|  | 1 | 'aged'/exp |  |
|  | 2 | 'geriatrics'/exp |  |
|  | 3 | 'geriatric nursing'/de OR 'psychogeriatric nursing'/de |  |
|  | 4 | elder*:ti,ab,kw OR eldest:ti,ab,kw OR frail*:ti,ab,kw OR geriatri*:ti,ab,kw OR 'old age':ti,ab,kw OR 'older adult*':ti,ab,kw OR 'older age':ti,ab,kw OR 'older female*':ti,ab,kw OR 'older male*':ti,ab,kw OR 'older man':ti,ab,kw OR 'older men':ti,ab,kw OR 'older patient*':ti,ab,kw OR 'older people*':ti,ab,kw OR 'older person*':ti,ab,kw OR 'older population':ti,ab,kw OR 'older subject*':ti,ab,kw OR 'older woman':ti,ab,kw OR 'older women':ti,ab,kw OR 'oldest old':ti,ab,kw OR senior*:ti,ab,kw OR senium:ti,ab,kw OR 'very old':ti,ab,kw |  |
|  | 5 | #1 OR #2 OR #3 OR #4 |  |
| Diet intervention | | | |
|  | 6 | 'diet'/exp |  |
|  | 7 | 'diet therapy'/exp |  |
|  | 8 | diet*:ti,ab,kw OR atherogenic:ti,ab,kw OR 'carbohydrate restricted':ti,ab,kw OR 'low carbohydrate':ti,ab,kw OR 'high protein low carb*':ti,ab,kw OR 'carbohydrate restricted high protein':ti,ab,kw OR 'south beach':ti,ab,kw OR atkins:ti,ab,kw OR ketogenic:ti,ab,kw OR keto:ti,ab,kw OR 'fat restricted':ti,ab,kw OR 'low fat':ti,ab,kw OR 'fat free':ti,ab,kw OR 'gluten free':ti,ab,kw OR 'high fat':ti,ab,kw OR 'high protein':ti,ab,kw OR mediterranean:ti,ab,kw OR paleolithic:ti,ab,kw OR 'stone age':ti,ab,kw OR paleo:ti,ab,kw OR caveman:ti,ab,kw OR 'hunter gatherer':ti,ab,kw OR 'protein restricted':ti,ab,kw OR 'low protein':ti,ab,kw OR 'protein free':ti,ab,kw OR 'sodium restricted':ti,ab,kw OR 'low sodium':ti,ab,kw OR 'low salt':ti,ab,kw OR 'salt free':ti,ab,kw OR vegetarian*:ti,ab,kw OR 'plant based':ti,ab,kw OR macrobiotic:ti,ab,kw OR vegan*:ti,ab,kw |  |
|  | 9 | ((food* OR nutrit* OR eat*) NEAR/2 (pattern* OR habit* OR intervent* OR therap*)):ti,ab,kw |  |
|  | 10 | #6 OR #7 OR #8 OR #9 |  |
| Working memory/short term memory | | | |
|  | 11 | 'memory'/de |  |
|  | 12 | 'working memory'/de |  |
|  | 13 | 'short term memory'/de |  |
|  | 14 | 'memory test'/exp |  |
|  | 15 | 'executive function'/exp |  |
|  | 16 | 'executive function test'/exp |  |
|  | 17 | memory:ti,ab,kw OR 'executive function':ti,ab,kw |  |
|  | 18 | 'embedded figures test':ti,ab,kw OR 'figural fluency test':ti,ab,kw OR 'rey osterrieth complex figure test':ti,ab,kw OR 'rod and frame test':ti,ab,kw OR 'simon task':ti,ab,kw OR 'stroop test':ti,ab,kw OR 'tower of london test':ti,ab,kw OR 'trail making test':ti,ab,kw OR 'wisconsin card sorting test':ti,ab,kw OR 'process dissociation procedure':ti,ab,kw OR 'buschke selective reminding test':ti,ab,kw OR 'california verbal learning test':ti,ab,kw OR 'hopkins verbal learning test':ti,ab,kw OR 'rey auditory verbal learning test':ti,ab,kw OR 'word list recall':ti,ab,kw |  |
|  | 19 | 'immediate recall*':ti,ab,kw OR 'cued recall':ti,ab,kw OR 'serial recall':ti,ab,kw |  |
|  | 20 | #11 OR #12 OR #13 OR #14 OR #15 OR #16 OR #17 OR #18 OR #19 |  |
| Combined sets | | | |
|  | 21 | #5 AND #10 AND #20 |  |
| Exclusion of conference abstracts | | | |
|  | 22 | #5 AND #10 AND #20 NOT [conference abstract]/lim |  |
| Limits: 2002-, english language | | | |
|  | 23 | #5 AND #10 AND #20 NOT [conference abstract]/lim AND [2002-2024]/py |  |
|  | 24 | #5 AND #10 AND #20 NOT [conference abstract]/lim AND [2002-2024]/py AND [english]/lim | 1876 |

Web of Science Core Collection (Clarivate)

Editions =
- WOS.SCI: 1975 to 2024
- WOS.AHCI: 1975 to 2024
- WOS.ESCI: 2019 to 2024
- WOS.ISTP: 1990 to 2024
- WOS.SSCI: 1975 to 2024
- WOS.ISSHP: 1990 to 2024

| Search terms | | | Total number of results |
| --- | --- | --- | --- |
| Elderly | | | |
|  | 1 | TS=(elder* or eldest or frail* or geriatri* or "old age" or "older adult*" or "older age" or "older female*" or "older male*" or "older man" or "older men" or "older patient*" or "older people*" or "older person*" or "older population" or "older subject*" or "older woman" or "older women" or "oldest old" or senior* or senium or "very old") |  |
| Diet intervention | | | |
|  | 2 | TS=(diet* or atherogenic or "carbohydrate restricted" or "low carbohydrate" or "high protein low carb*" or "carbohydrate restricted high protein" or "south beach" or atkins or ketogenic or "keto" or "fat restricted" or "low fat" or "fat free" or "gluten free" or "high fat" or "high protein" or mediterranean or paleolithic or "stone age" or "paleo" or caveman or "hunter gatherer" or "protein restricted" or "low protein" or "protein free" or "sodium restricted" or "low sodium" or "low salt" or "salt free" or vegetarian* or "plant based" or macrobiotic or vegan*) |  |
|  | 3 | TS=((food* or nutrit* or eat*) NEAR/1 (pattern* or habit* or intervent* or therap*)) |  |
|  | 4 | #2 OR #3 |  |
| Working memory/short term memory | | | |
|  | 5 | TS=("executive function" or memory or "embedded figures test" or "figural fluency test" or "Rey Osterrieth complex figure test" or "rod and frame test" or "Simon task" or "Stroop test" or "tower of London test" or "trail making test" or "Wisconsin Card Sorting Test" or "process dissociation procedure" or "Buschke selective reminding test" or "California verbal learning test" or "Hopkins verbal learning test" or "Rey auditory verbal learning test" or "word list recall" or "immediate recall*" or "cued recall" or "serial recall") |  |
| Combined Sets | | | |
|  | 6 | #1 AND #4 AND #5 |  |
| Limits: Timespan: 2002-01-01 to 2024-12-31 (Index Date), English | | | |
|  | 7 | #1 AND #4 AND #5 and English (Languages) | 2025 |

Cochrane library

| Search terms | | | Total number of results |
| --- | --- | --- | --- |
| Aged | | | |
|  | 1 | MeSH descriptor: [Aged] explode all trees |  |
|  | 2 | MeSH descriptor: [Geriatrics] explode all trees |  |
|  | 3 | MeSH descriptor: [Cognitive Aging] this term only |  |
|  | 4 | MeSH descriptor: [Geriatric Nursing] this term only |  |
|  | 5 | (elder* or eldest or frail* or geriatri* or "old age" or older NEXT adult* or "older age" or older NEXT female* or older NEXT male* or "older man" or "older men" or older NEXT patient* or older NEXT people* or older NEXT person* or "older population" or older NEXT subject* or "older woman" or "older women" or "oldest old" or senior* or senium or "very old"):ti,ab,kw |  |
|  | 6 | #1 or #2 or #3 or #4 or #5 |  |
| Diet intervention | | | |
|  | 7 | MeSH descriptor: [Diet] explode all trees |  |
|  | 8 | MeSH descriptor: [Diet Therapy] explode all trees |  |
|  | 9 | MeSH descriptor: [] explode all trees and with qualifier(s): [diet therapy - DH] |  |
|  | 10 | MeSH descriptor: [Feeding Behavior] explode all trees |  |
|  | 11 | (diet* or atherogenic or "carbohydrate restricted" or "low carbohydrate" or "high protein low carb" or "high protein low carbs" or "carbohydrate restricted high protein" or "south beach" or atkins or ketogenic or keto or "fat restricted" or "low fat" or "fat free" or "gluten free" or "high fat" or "high protein" or mediterranean or paleolithic or "stone age" or paleo or caveman or "hunter gatherer" or "protein restricted" or "low protein" or "protein free" or "sodium restricted" or "low sodium" or "low salt" or "salt free" or vegetarian* or "plant based" or macrobiotic or vegan*):ti,ab,kw |  |
|  | 12 | ((food* or nutrit* or eat*) NEAR/2 (pattern* or habit* or intervent* or therap*)):ti,ab,kw |  |
|  | 13 | #7 or #8 or #9 or #10 or #11 or #12 |  |
| Working memory/short term memory | | | |
|  | 14 | MeSH descriptor: [Memory] this term only |  |
|  | 15 | MeSH descriptor: [Memory, Short-Term] this term only |  |
|  | 16 | MeSH descriptor: [Executive Function] this term only |  |
|  | 17 | MeSH descriptor: [Neuropsychological Tests] this term only |  |
|  | 18 | MeSH descriptor: [Memory and Learning Tests] explode all trees |  |
|  | 19 | MeSH descriptor: [Wisconsin Card Sorting Test] this term only |  |
|  | 20 | (memory):ti,ab,kw |  |
|  | 21 | ("executive function"):ti,ab,kw |  |
|  | 22 | ("embedded figures test" or "figural fluency test" or "Rey Osterrieth complex figure test" or "rod and frame test" or "Simon task" or "Stroop test" or "tower of London test" or "trail making test" or "Wisconsin Card Sorting Test" or "process dissociation procedure" or "Buschke selective reminding test" or "California verbal learning test" or "Hopkins verbal learning test" or "Rey auditory verbal learning test" or "word list recall"):ti,ab,kw |  |
|  | 23 | (immediate NEXT recall* or "cued recall" or "serial recall"):ti,ab,kw |  |
|  | 24 | #14 or #15 or #16 or #17 or #18 or #19 or #20 or #21 or #22 or #23 |  |
| Combined Sets | | | |
|  | 25 | #6 and #13 and #24 |  |
| Limits: Years: 2002-2022 | | | |
|  | 26 | 25 | 806 |

**Supplementary Material S2: Studies Excluded After Full Text Screening**

| **Title** | **Authors** | **PMID** | **Reason for exclusion** |
| --- | --- | --- | --- |
| Efficacy of Dietary Intervention with Group Activities on Dietary Intakes, Frailty Status, and Working Memory: A Cluster-Randomized Controlled Trial in Community Strongholds | S.-Y. Wu; Y.-Y. Cheng; H.-Y. Chang; P.-H. Wang; I. C. Hsieh; N.-H. Yeh; K.-C. Huang; W.-H. Pan | **37111195** | Cluster-randomized trial; difficult to determine the age range for inclusion |
| The Impact of Pandemic-Related Restrictions on Dementia Risk Factors in Older Adults | T. Simone; C. Peltz; D. E. Rosenberg; D. E. Barnes; L. E. Fleckenstein; S. Dublin; K. Yaffe | **37930366** | Not an intervention (although cohort was recruited from the intervention) |
| Observed Improvement in Cognition During a Personalized Lifestyle Intervention in People with Cognitive Decline | H. Sandison; N. G. L. Callan; R. V. Rao; J. Phipps; R. Bradley | **37355891** | Uncontrolled, pragmatic trial design; inclusion criteria for participants was 45 years and older |
| Physical exercise in MCI elderly promotes reduction of pro-inflammatory cytokines and improvements on cognition and BDNF peripheral levels | C. M. Nascimento; J. R. Pereira; L. P. de Andrade; M. Garuffi; L. L. Talib; O. V. Forlenza; J. M. Cancela; M. R. Cominetti; F. Stella | **25212919** | Physical exercise intervention only (not diet) |
| The Effect of a Mediterranean Diet With or Without Calorie Restriction on Cognition, Lifestyle, and Metabolic Health of Obese, Predominately Non-Hispanic Black Older Adults | A. McLeod; L. Tussing-Humphreys; M. Antonic; M. Berbaum; L. Blumstein; R. Dakers-Jones; A. Karstens; M. Lamar; N. OjiHemphill; L. Restrepo; et al. | NA | Conference proceeding |
| Effect of New Complete Dentures and Simple Dietary Advice on Cognitive Screening Test among Edentulous Older Adults: A Randomized Controlled Trial | Y. Komagamine; H. Suzuki; M. Iwaki; S. Minakuchi; M. Kanazawa | **37510823** | Not a lifestyle multimodal intervention |
| Modified Korean MIND Diet: A Nutritional Intervention for Improved Cognitive Function in Elderly Women through Mitochondrial Respiration, Inflammation Suppression, and Amino Acid Metabolism Regulation | E. Y. Kang; D. Y. Kim; H. K. Kim; W. S. Shin; Y. S. Park; T. H. Kim; W. Kim; L. Cao; S. Lee; G. Gang; M. Shin; J. M. Kim; G. Go | **37650267** | Not a controlled intervention |
| A randomized feasibility trial of the modified Atkins diet in older adults with mild cognitive impairment due to Alzheimer's disease | A. Buchholz; P. Deme; J. F. Betz; J. Brandt; N. Haughey; M. C. Cervenka | **38505743** | Pilot study, but based on RCT. Recruited from AD patient registry |
| Longer Term Effects of Diet and Exercise on Neurocognition: 1‐Year Follow‐up of the ENLIGHTEN Trial | J. A. Blumenthal; P. J. Smith; S. Mabe; A. Hinderliter; K. Welsh‐Bohmer; J. N. Browndyke; P. M. Doraiswamy; P. Lin; W. E. Kraus; J. R. Burke; et al. | **31755550** | Participants age = 55yr or older |
| Virgin olive oil supplementation and long-term cognition: the PREDIMED-NAVARRA randomized, trial. | E.H. Martinez-Lapiscina, P. Clavero, E. Toledo, B. San Julian, A. Sanchez-Tainta, D. Corella, et al. | **23732551** | Participants were younger than 60 yrs |
| Mediterranean diet improves cognition: the PREDIMED-NAVARRA randomised trial | E.H. Martinez-Papiscina, P. Clavero, E. Toledo, R. Estruch, J. Salas-Salvado, B. San Julian, A. Sanchez-Tainta, et al. | **23670794** | Participants were younger than 60 yrs |
| Dietary protein, carbohydrate, and fat enhance memory performance in the healthy elderly | R.J. Kaplan, C.E. Greenwood, G. Winocur, T.M. Wolever | **11684539** | Macronutrient suppl does not meet inclusion criteria for whole-diet intervention |
| Effects of a Primary Care-Based Multifactorial Intervention on Physical and Cognitive Function in Frail, Elderly Individuals: a Randomized Controlled Trial | L. Romera-Liebana, F. Orfila, J. Maria Segura, J. Real, M. Lluisa Fabra, M. Möller, et al. | **29346524** | Hyperproteic shake does not meet inclusion criteria for whole-diet intervention |
| Effects of Fruit and Vegetable-Based Nutraceutical on Cognitive Function in a Healthy Population: Placebo-Controlled, Double-Blind, and Randomized Clinical Trial | J.A. Carillo, R. Arcusa, M.P. Zafrilla, J. Marhuenda | 33467530 | Participants were younger than 60 yrs (18-65) and not a whole-diet intervention |
| Caloric restriction improves memory in elderly humans | A.V. Witte, M. Fobker, R. Gellner, S. Knecht, A. Flöel | **19171901** | Particpants were younger than 60 years (50-80 yrs) |
| The effect of spermidine on memory performance in older adults at risk for dementia: A randomized controlled trial | M. Wirth, G. Benson, C. Schwarz, T. Köbe, U. Grittner, D. Schmitz, S.J. Sigist, et al. | **30388439** | Supplementation (plant extract, rich in spirimidine) |
| Promotion of Healthy Aging Within a Community Center Through Behavior Change: Health and Fitness Findings From the AgeWell Pilot Randomized Controlled Trial. | J.M. Thorn, S.M. Nelis, J.K. Cooney, J.V. Hindle, I.R. Jones, L. Clare | **32781433** | Participant age under 60 |
| Nutraceutical intervention improves older adults' cognitive functioning. | B.J. Small, K.S. Rawson, C Martin, S.L. Eisel, C.D. Sandberg, C.L. McEvoy, P.R. Sanberg, et al. | **24134194** | Supplement, not whole-diet intervention |
| Blended home-based exercise and dietary protein in community-dwelling older adults: a cluster randomized controlled trial | J. van den Helder, S. Mehra, C. van Dronkelaar, G. ter Riet, M. Tieland, B. Visser, B.J.A. Kröse, et al. | **33103379** | Paricipants under 60 years |
| Building research in diet and cognition: The BRIDGE randomized controlled trial | L. Tussing-Humphreys, M. Lamar, J. A. Blumenthal, M. Babyak, G. Fantuzzi, L. Blumstein, et al. | **28600159** | Conducted in obese individuals |
| Cognitive Effects of Multi-Domain Interventions Among Pre-Frail and Frail Community-Living Older Persons: Randomized Controlled Trial | T. P. Ng, L. H. A. Ling, L. Feng, M. S. Z. Nyunt, L. Feng, M. Niti, et al. | **29069291** | Conducted specifically in frail and pre-frail older adults |
| Greek High Phenolic Early Harvest Extra Virgin Olive Oil Reduces the Over-Excitation of Information-Flow Based on Dominant Coupling Mode (DoCM) Model in Patients with Mild Cognitive Impairment: An EEG Resting-State Validation Approach | S. I. Dimitriadis, C. Lyssoudis, A. C. Tsolaki, E. Lazarou, M. Kozori and M. Tsolaki | **34308906** | Not appropriate control; investigating effects of extra virgin olive oil (food supplement) |
| The impact of protein supplementation on cognitive performance in frail elderly | N. L. van der Zwaluw, O. van de Rest, M. Tieland, J. J. Adam, G. J. Hiddink, L. J. C. van Loon, et al. | **24045855** | Protein supplement |
| Longer-term soy nut consumption improves cerebral blood flow and psychomotor speed: results of a randomized, controlled crossover trial in older men and women | J. P. D. Kleinloog, L. Tischmann, R. P. Mensink, T. C. Adam and P. J. Joris | **34510189** | Food supplement |
| Differential Effects of Meal Challenges on Cognition, Metabolism, and Biomarkers for Apolipoprotein E e4 Carriers and Adults with Mild Cognitive Impairment | A. J. Hanson, J. L. Bayer, L. D. Baker, B. Cholerton, B. VanFossen, E. Trittschuh, et al. | **26401941** | Included participants with early AD |
| Caloric Restriction in Older Adults-Differential Effects of Weight Loss and Reduced Weight on Brain Structure and Function | K. Prehn, R. Jumpertz von Schwartzenberg, K. Mai, U. Zeitz, A. V. Witte, D. Hampel, et al. | **26838769** | Mean age 60.5 yrs; some participants below 60 years |
| Modified ketogenic diet is associated with improved cerebrospinal fluid biomarker profile, cerebral perfusion, and cerebral ketone body uptake in older adults at risk for Alzheimer's disease: a pilot study | B. J. Neth, A. Mintz, C. Whitlow, Y. Jung, K. Solingapuram Sai, T. C. Register, et al. | **31757576** | No inclusion criteria for age |
| Cognitive Benefit of a Multidomain Intervention for Older Adults at Risk of Cognitive Decline: A Cluster- Randomized Controlled Trial | X. M. Liu; Z. Y. Ma; X. Y. Zhu; Z. W. Zheng; J. Li; J. N. Fu; Q. Shao; X. Y. Han; Z. H. Wang; X. N. Wang; Z. X. Yin; C. X. Qiu | **36414488** | Cluster-randomized trial |
